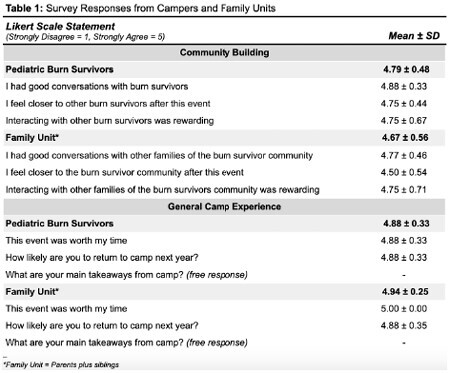# 727 Medical Student-Run Camp for Pediatric Burn Survivors: Patient, Family, and Volunteer Impact

**DOI:** 10.1093/jbcr/irae036.270

**Published:** 2024-04-17

**Authors:** Devon O'Brien, Joseph Maestas, Erin E Ross, Michael I Kim, Abigail Song, Justin Gillenwater

**Affiliations:** Keck School of Medicine of USC, South Pasadena, CA; Keck School of Medicine of USC, Los Angeles, CA; Keck Medicine of USC, Los Angeles, CA; Keck School of Medicine of USC, South Pasadena, CA; Keck School of Medicine of USC, Los Angeles, CA; Keck Medicine of USC, Los Angeles, CA; Keck School of Medicine of USC, South Pasadena, CA; Keck School of Medicine of USC, Los Angeles, CA; Keck Medicine of USC, Los Angeles, CA; Keck School of Medicine of USC, South Pasadena, CA; Keck School of Medicine of USC, Los Angeles, CA; Keck Medicine of USC, Los Angeles, CA; Keck School of Medicine of USC, South Pasadena, CA; Keck School of Medicine of USC, Los Angeles, CA; Keck Medicine of USC, Los Angeles, CA; Keck School of Medicine of USC, South Pasadena, CA; Keck School of Medicine of USC, Los Angeles, CA; Keck Medicine of USC, Los Angeles, CA

## Abstract

**Introduction:**

Burn day camp participation can improve self-esteem, communication skills, and depression/anxiety symptoms; however, survivors of racial minority groups have historically derived fewer benefits. Community building and resource access outcomes among burn camp participants and the potential impact of burn camp volunteerism on medical students have not been studied. Here, we assess the success of a medical student-run burn camp, serving predominantly minority groups, in building burn survivor community, improving resource access, and impacting medical student volunteers.

**Methods:**

School-aged burn survivors (6-17 years) treated at the LA General Regional Burn Center and their families were invited to a day camp involving community building and mentorship activities hosted by medical student volunteers in October 2022. Children participated in arts and crafts, science experiments, and field games with student volunteers; parents engaged with burn community resources including physical/occupational therapy education, scar management education from burn unit registered nurses, and a local non-profit that supports survivors. Camp participants and medical students each completed a post-camp survey of quantitative and qualitative questions (Tables 1-2). Anonymized camper surveys were aggregated into summary variables for community building and general camp experience, and analyzed with an inter-group factor (child/parents plus siblings).

**Results:**

Camp participants totaled 44 people from eight family units (11 pediatric burn survivors, 21 siblings, and 12 parents). Most survivors (n=10, 90.9%) were from racial or ethnic minority groups. All survivors reported increased burn community building (4.79/5) and all eight families reported increased community building (4.67/5). Five families (62.5%) noted the value of connecting with community resources available at the camp. Among 60 medical student volunteers, 15 (25%) completed the post-camp survey; all respondents agreed they could improve the lives of children with burn injuries (4.80/5) and all agreed they would likely volunteer again (4.80/5). Twelve (80%) volunteers specifically noted they valued learning more about the unique experiences and resilience of the burn survivor community.

**Conclusions:**

Student-run day camps for pediatric burn survivors and their families can foster relationships between survivors, integrate families into the existing survivor community, and provide access to burn community resources. Student volunteers learned about the experience of pediatric burn survivors and their unique needs and resilience.

**Applicability of Research to Practice:**

Medical student-run burn camps affiliated with urban burn centers may foster connections for patients with less resources to the burn survivor community. Self-directed service may improve medical student attitudes towards the burn survivor experience and promote future service for this vulnerable population.